# A Case Report of Piriformis Syndrome With Axial Spondyloarthritis in a Young Male Patient Presenting With Buttock Pain

**DOI:** 10.7759/cureus.101642

**Published:** 2026-01-15

**Authors:** Hanxue Li, Jinmei Su

**Affiliations:** 1 Department of Rheumatology and Clinical Immunology, Peking Union Medical College Hospital, Chinese Academy of Medical Sciences, Peking Union Medical College, Beijing, CHN

**Keywords:** axial spondyloarthritis, bone marrow edema, buttock pain, mechanical stress, piriformis syndrome (ps)

## Abstract

This study explores the clinical challenges in differentiating axial spondyloarthritis (axSpA) from piriformis syndrome (PS). A male patient in his 20s presented with lumbar and gluteal pain after weight-bearing. Initially diagnosed with PS, his symptoms persisted despite nonpharmacological treatment. After a thorough assessment, he was ultimately diagnosed with axSpA. This case underscores the role of imaging in differentiating axSpA from transient bone marrow edema and provides insights into distinguishing axSpA from PS in clinical practice to provide guidance for accurate diagnosis and treatment in similar complex cases.

## Introduction

Low back pain is the most common cause of diminished work capability and poor quality of life, with a global incidence of 3,176.6 per 100,000 and a prevalence of 7,463.1 per 100,000 [1]. Axial spondyloarthritis (axSpA) is a leading cause of chronic low back pain, primarily characterized by inflammation of the sacroiliac joints and spine. Its global prevalence ranges from 0.3% to 1.4% [2] and is strongly associated with human leukocyte antigen-B27 (HLA-B27). Typical axSpA symptoms include alternating buttock pain and inflammatory low back pain that worsens at rest but improves with movement. Piriformis syndrome (PS) is a type of sciatica caused by the piriformis muscle compressing the sciatic nerve, which typically manifests as buttock pain. PS is frequently misunderstood for axSpA. PS is the second common musculoskeletal abnormality among people with suspected sacroiliitis; however, it has no definitive magnetic resonance imaging (MRI) evidence (4.2%) [3].

This report describes a case of a young adult with low back pain and hip discomfort as the primary manifestations of PS, as well as axSpA activity during heavy and weight-bearing labor. This case report explores two prevalent issues in clinical work. First, lower back pain can be caused by exercise or heavy weight-bearing physical labor, and an MRI can reveal bone marrow edema (BME) [4]. It will address how to determine whether the MRI manifestation is temporary or caused by axSpA. Second, it will also address how to detect PS and axSpA in clinical cases to provide more accurate treatment.ff

## Case presentation

**Figure 1:**
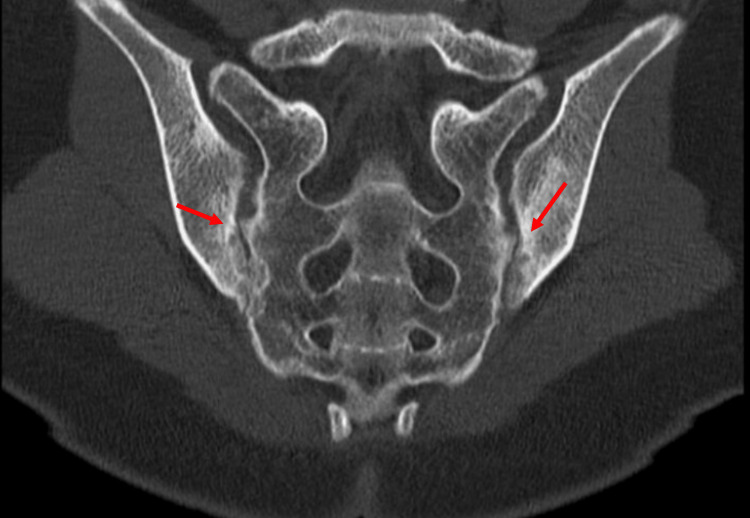
Computed tomography of the sacroiliac joint The arrow shows roughened and sclerotic joint surfaces, several tiny hypodense foci beneath the joint surfaces, and narrowing of the bilateral joint spaces

This case report involves a man in his mid-20s presented with intermittent low back pain, which began in January 2024. The pain was mild, and he did not seek medical treatment during the initial days. In July 2024, he started working that involved heavy lifting, which subsequently worsened his back pain, accompanied by morning stiffness and alternating buttock pain. In January 2025, severe pain prompted him to visit the hospital near home. Based on his medical history, symptoms, and physical examination (pain during deep palpation of the piriformis muscle), he was diagnosed with PS, and rest and physical treatment were advised. In March 2025, the patient presented to Peking Union Medical College Hospital with worsening discomfort. Physical examination revealed that the piriformis muscle provocation test of the patient was positive. Laboratory tests revealed significantly high C-reactive protein (CRP) levels, erythrocyte sedimentation rate (ESR), and HLA-B27 positivity. MRI of the sacroiliac joint revealed several regions of BME in the sacroiliac joints. Further computed tomography of the sacroiliac joint revealed roughened and sclerotic joint surfaces, several tiny hypodense foci beneath the joint surfaces, and narrowing of the bilateral joint spaces (Figure 1).

**Figure 2:**
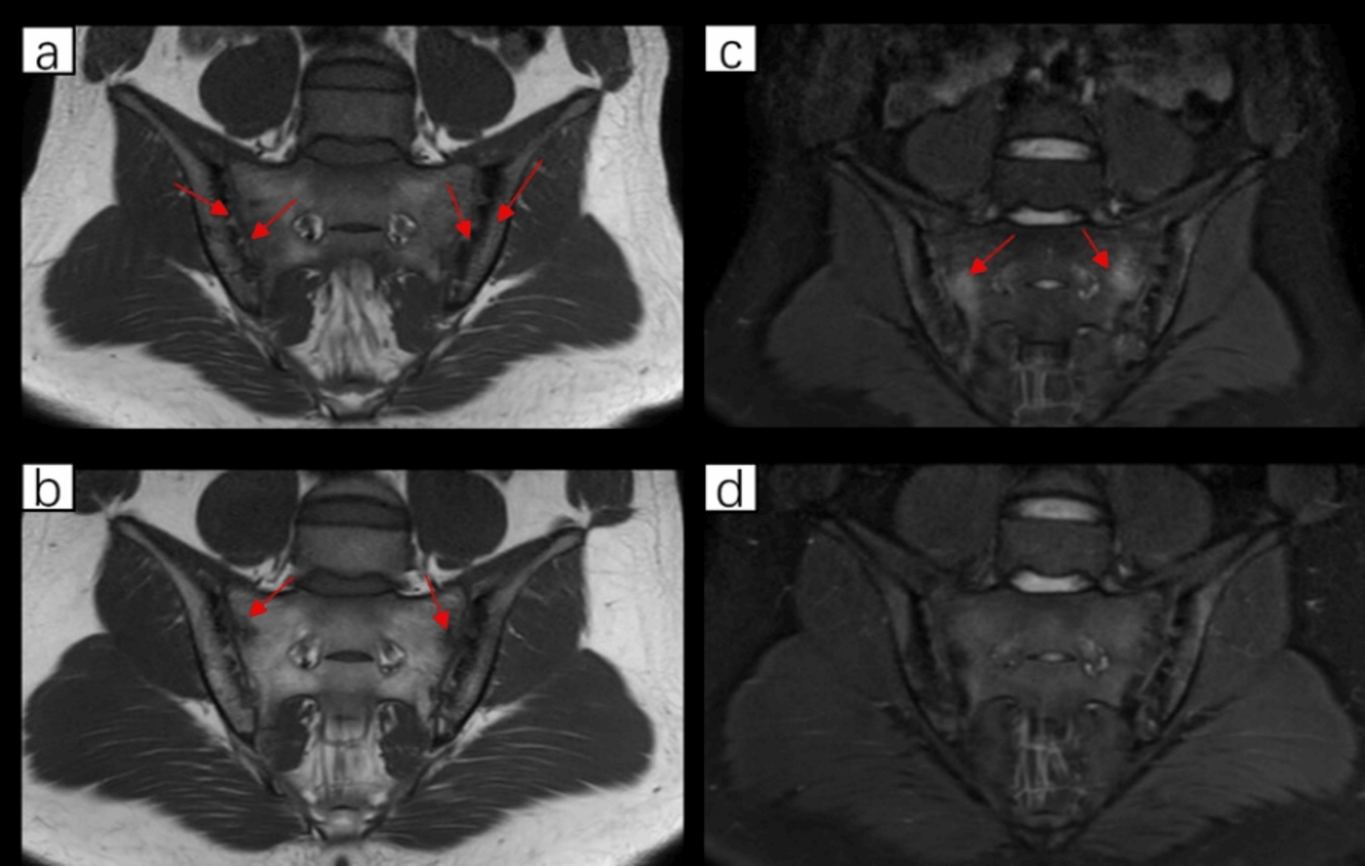
MRI of the sacroiliac joint before and after treatment (a) T1WI, March 2025; bilateral sacroiliac joint surface roughness with multiple worm-eaten-like changes beneath the articular surfaces, accompanied by narrowing of the joint space, is marked with arrows. (b) T1WI, July 2025; the arrow indicates fat metaplasia/depositopn. (c) T2 fat suppression sequence, March 2025; the arrow indicates bone marrow edema. (d) T2 fat suppression sequence, July 2025; the bone marrow edema has disappeared T1WI: T1-weighted imaging

According to the Assessment of SpondyloArthritis International Society 2009 classification criteria for axSpA [5], the diagnosis of axSpA was made based on the patient's clinical features (onset age less than 45 years, low back pain lasting three months or longer, positive HLA-B27, and definitive sacroiliitis on imaging). Combined with the patient's initial diagnosis of PS at another hospital, the patient was ultimately diagnosed with axSpA accompanied by PS. Although the lumbar and buttock discomfort was substantially alleviated by the administration of oral loxoprofen, the patient's CRP and ESR levels were still high after about four weeks. The recommended treatment was an interleukin-17 (IL-17) inhibitor at 150 mg weekly (modified to every four weeks after five weeks) in combination with celecoxib 200 mg twice daily for anti-inflammatory and pain relief. This regimen largely relieved the patient’s pain, and follow-up laboratory tests indicated that inflammatory markers had returned to near-normal levels (Table 1). Three months after treatment, an MRI of the sacroiliac joint demonstrated a significant reduction in acute inflammatory signals beneath both sacroiliac joint surfaces; a comparison of the MRI before and after treatment is shown in Figure 2. The treatment was successful, and IL-17 inhibitor therapy remains in use with ongoing follow-up.

**Table 1:** The patient’s laboratory test results and treatment

Parameter	2025/3	2025/4	2025/7	Reference ranges
Treatment	-	Loxoprofen	Interleukin-17 inhibitor and celecoxib	-
Human leukocyte antigen-B27	Positive	-	-	Negative
C-reactive protein (mg/L)	82.41	45.28	1.78	0-8
Erythrocyte sedimentation rate (mm/hour)	114	57	1	0-15
White blood cell (×10^9^/L)	7.92	4.67	8.30	3.5-9.5
Hemoglobin (g/L)	127	130	150	120-160 (male)
Platelet (×10^9^/L)	370	238	266	100-350

## Discussion

This case illustrates the diagnostic challenge of distinguishing PS from axSpA in young patients presenting with exertional buttock pain. The initial, isolated diagnosis of PS, later revised to concomitant axSpA, highlights a common clinical pitfall and underscores the critical importance of a comprehensive diagnostic approach when inflammatory features are present or symptoms are mechanically exacerbated. During the diagnostic workup, common mimics such as lumbar disc herniation were considered. However, the patient’s inflammatory symptoms and the clear involvement of the sacroiliac joint and piriformis region made a primary lumbar cause unlikely. Therefore, dedicated lumbar spine imaging was not necessary. Additionally, piriformis infection was considered unlikely due to the chronic and intermittent nature of the symptoms, the absence of systemic signs of infection, and consistently normal white blood cell counts.

Initially, MRI can detect sacroiliitis after strenuous physical labor or heavy lifting. Thus, a crucial clinical issue is how to distinguish axSpA from transient sacroiliitis resulting from mechanical stress. The principal distinctions lie in the clinical presentation and, crucially, in the imaging profile (Table 2). Stress-related BME is typically focal and transient, without the erosions or fat metaplasia characteristic of chronic axSpA, where BME is more diffuse and associated with these specific structural changes [2,4].

**Table 2:** Differences between sacroiliitis caused by mechanical stress injury and axial spondyloarthritis BME: bone marrow edema

Characteristics	Axial spondyloarthritis	Sacroiliitis caused by mechanical stress injury
Characteristics of pain	Pain may reduce after activity but intensify with rest, often accompanied by morning stiffness [2]	Pain is related to exercise intensity; it worsens during activity and relieves with rest [6]
Imaging characteristics [2,4]		
BME	Diffuse, involving multiple quadrants, with no specific preferred region, is typically more widespread and severe	Highly concentrated in the posterior inferior iliac spine and anterior superior sacrum
Fat metaplasia/depositopn	Common, resulting from chronic inflammation or postrepair processes, often appearing in areas where BME has resolved (“reparative” changes)	Relatively uncommon, with no spatial correlation to BME
Erosion	Common and highly specific, it serves as a key diagnostic basis	Relatively uncommon
Ankylosis	Late-stage features, manifested as bone bridging or joint fusion	Almost nonexistent

Additionally, mechanical stress injury is a key factor in axSpA onset and a major driver of disease progression. Mechanical stress activates inflammatory and bone-remodeling pathways, including mitogen-activated protein kinase (extracellular signal-regulated kinase 1/2), tumor necrosis factor-alpha, and transforming growth factor-beta, leading to lesions such as enthesitis, synovitis, and bone erosion [7]. Clinical studies have further confirmed that compared with nonmanual workers, patients with axSpA engaged in physical labor exhibit more severe radiographic damage and faster radiographic progression [8,9]. Therefore, for individuals at risk of axSpA, such as those with a family history, avoiding hard physical labor or strenuous exercise can prevent triggering axSpA onset. Similarly, for patients with axSpA, avoiding heavy labor or strenuous exercise can help prevent disease progression.

Misdiagnosis is common because the overlapping symptom of buttock pain means PS is a common diagnostic mimic of axSpA. Distinguishing between them requires a systematic evaluation beyond history alone, integrating targeted physical examination, laboratory tests, and imaging. The key differential features are summarized in Table 3 [2,10]. In brief, axSpA is characterized by systemic inflammatory signs (elevated CRP/ESR, high HLA-B27 positivity), inflammatory back pain, and potentially extra-articular manifestations [2]. Imaging, particularly MRI, is pivotal for revealing characteristic sacroiliitis (BME, erosions, and fat metaplasia) [2]. In contrast, PS is primarily a clinical diagnosis of exclusion. Laboratory tests are typically normal, and while imaging may rule out other pathologies or show piriformis muscle changes, it lacks diagnostic specificity for PS itself [10]. Provocative physical maneuvers differ, targeting the sacroiliac joint in axSpA, for example, flexion, abduction, and external rotation maneuver [11], vs. reproducing sciatic nerve irritation by the piriformis muscle in PS, for example, flexion, adduction, and internal rotation maneuver [10].

**Table 3:** Differential diagnosis of axial spondyloarthritis and piriformis syndrome FABER: flexion, abduction, and external rotation; FAIR: flexion, adduction, and internal rotation; HLA-B27: human leukocyte antigen-B27

Parameter	Axial spondyloarthritis [2]	Piriformis syndrome [10]
Clinical manifestations	Inflammatory low back pain (e.g., morning stiffness and relief after activity), alternating buttock pain extra-articular manifestations (e.g., uveitis, psoriasis, and inflammatory bowel disease)	Pain in the buttocks radiating down the lower limbs, worsening with prolonged sitting
Physical examination	Sacroiliac joint compression pain, FABER test positive, restricted spinal mobility	Deep palpation and provocation tests were positive (FAIR test)
Laboratory tests	HLA-B27 positivity rate: 70%-90%, C-reactive protein/erythrocyte sedimentation rate are often elevated	The HLA-B27 positivity rate is similar to that in the general population, approximately 10%
Imaging examinations	X-rays reveal structural damage (such as sclerosis, erosion, and joint space narrowing). Magnetic resonance imaging can assist in the early diagnosis of axial spondyloarthropathy, demonstrating active inflammation (such as bone marrow edema) and structural damage (such as fat deposition and bone erosion)	The primary value lies in ruling out other causes; in some patients, imaging studies may reveal piriformis muscle morphological abnormalities or signal changes

This case underscores the risk of an “inadequate initial assessment” when evaluating unilateral or bilateral buttock pain. The initial diagnosis of PS was reasonable given the presentation and positive provocation test. However, the persistence of symptoms and the presence of inflammatory clues (morning stiffness, symptom worsening with rest) were red flags warranting further investigation. A definitive diagnosis of axSpA was only achieved through systematic serological testing (CRP, ESR, and HLA-B27) and advanced imaging (MRI of sacroiliac joints). This sequence argues for a low threshold to perform these investigations in similar cases, particularly in young male patients with mechanical stress exposure.

**Figure 3:**
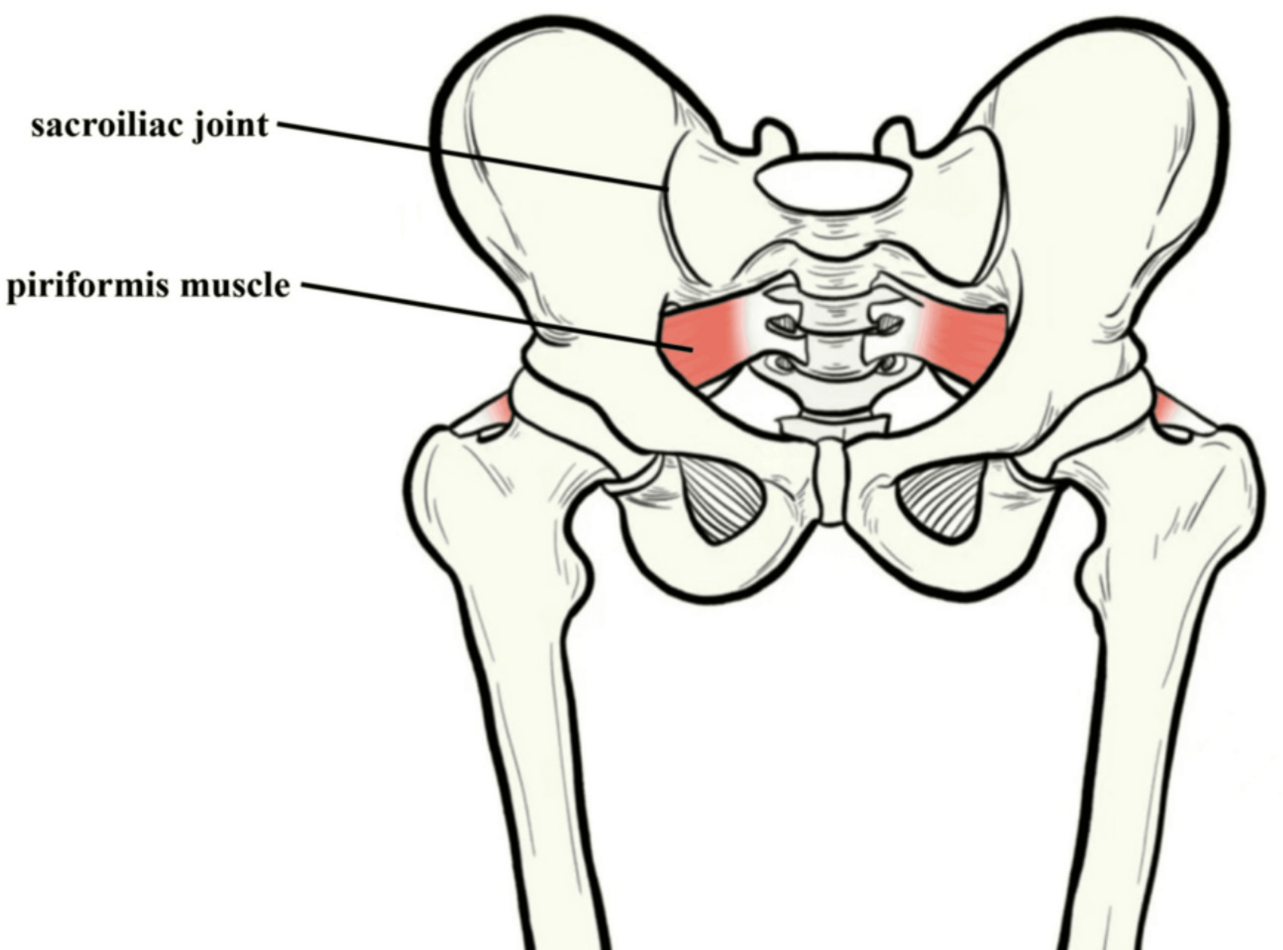
Anatomical location of the sacroiliac joint and piriformis muscle Image credit: This is an original image created by the author Hanxue Li using the Procreate app (Savage Interactive, Hobart, Australia)

Additionally, axSpA and PS may coexist. Approximately 10% of patients with axSpA exhibit radiographic evidence of PS, and this subgroup demonstrates higher disease activity based on the ankylosing spondylitis disease activity score calculated using CRP and more severe functional impairment based on the Bath ankylosing spondylitis functional index [12]. Ultrasound studies reveal that the piriformis muscle in patients with axSpA is often significantly thickened and increased in hardness, and these changes positively correlated with disease duration [13]. Accordingly, axSpA may induce pathological alterations in the piriformis muscle. The mechanism likely involves the proximity of the piriformis muscle to the sacroiliac joint (Figure 3). Consequently, sacroiliac joint inflammation can directly spread or trigger referred pain, manifesting as PS symptoms [14-16].

Treatment for spondyloarthritis is systemic and requires long-term standardized management based on guidelines. It emphasizes shared decision-making between physicians and patients, combining nonpharmacological interventions (such as exercise) with a stepwise medication strategy (escalating from nonsteroidal anti-inflammatory drugs (NSAIDs) to biologics) [17]. Medication should be individualized according to extra-articular manifestations. Although the patient's clinical symptoms improved after four weeks of NSAIDs, CRP and ESR remained elevated. Due to inadequate control of the patient's inflammation, the physician and patient jointly decided to initiate treatment with an IL-17 inhibitor based on guidelines [17]. The dosing regimen selected is the standard protocol for axSpA. Treatment for PS focuses on local conservative approaches, primarily involving medications for symptom relief, physical therapy for muscle stretching and relaxation, and mechanical correction [10]. Local injections may be used for difficult-to-treat cases, whereas surgery is rarely required.

When patients with axSpA demonstrate poor response to anti-inflammatory therapy, the coexistence of PS and spondyloarthritis should be considered. Treatment for patients with the coexistence of axSpA and PS is not a simple overlap of therapies. It should involve controlling systemic inflammation while adding localized interventions (e.g., physical therapy or injections) as needed.

This report addresses two practical questions raised in the Introduction section. First, regarding the differentiation of axSpA from mechanical sacroiliitis, key distinctions in pain patterns and, decisively, in MRI findings (Table 2) allow for this discrimination. Second, regarding the detection of PS and axSpA, a stepwise diagnostic algorithm integrating clinical suspicion, targeted physical exams, inflammatory markers, HLA-B27 status, and definitive MRI evidence is essential, as illustrated herein.

## Conclusions

This case presents a young man primarily presenting with low back pain, morning stiffness, and alternating buttock pain. Initially diagnosed with PS, he was later diagnosed with axSpA based on the HLA-B27 testing, inflammatory markers, and characteristic sacroiliac joint MRI findings. Thus, careful assessment of the source of pain is essential for patients with low back pain. Thorough physical examination, comprehensive serological testing, and MRI evaluation should be conducted to distinguish axSpA, thereby avoiding delayed diagnosis and treatment. Mechanical stress is one of the causative factors in axSpA. Conducting an MRI early in the disease course is recommended for patients with persistent low back pain under prolonged mechanical load. Mechanical stress can also hasten spondyloarthritis progression, often leading to more severe radiographic damage and functional impairment in physical laborers. Therefore, susceptible individuals or patients with axSpA should avoid weight-bearing occupations whenever possible to reduce the effect on the prognosis of spondyloarthritis.
