# Correction: A Case Report of Piriformis Syndrome With Axial Spondyloarthritis in a Young Male Patient Presenting With Buttock Pain

**DOI:** 10.7759/cureus.c446

**Published:** 2026-06-03

**Authors:** Hanxue Li, Jinmei Su

**Affiliations:** 1 Department of Rheumatology and Clinical Immunology, Peking Union Medical College Hospital, Chinese Academy of Medical Sciences, Peking Union Medical College, Beijing, CHN

Figure 2 has been updated to correct panels 2a and 2b. These panels now correctly show T1-weighted images.

